# A machine-learning model for reducing misdiagnosis in heparin-induced thrombocytopenia: A prospective, multicenter, observational study

**DOI:** 10.1016/j.eclinm.2022.101745

**Published:** 2022-11-24

**Authors:** Henning Nilius, Adam Cuker, Sigve Haug, Christos Nakas, Jan-Dirk Studt, Dimitrios A. Tsakiris, Andreas Greinacher, Adriana Mendez, Adrian Schmidt, Walter A. Wuillemin, Bernhard Gerber, Johanna A. Kremer Hovinga, Prakash Vishnu, Lukas Graf, Alexander Kashev, Raphael Sznitman, Tamam Bakchoul, Michael Nagler

**Affiliations:** a Department of Clinical Chemistry, Inselspital, Bern University Hospital, University of Bern, Bern, Switzerland; b Department of Medicine and Department of Pathology and Laboratory Medicine, University of Pennsylvania Perelman School of Medicine, Philadelphia, PA, USA; c Mathematical Institute, University of Bern, Bern, Switzerland; d Albert Einstein Center for Fundamental Physics and Laboratory for High Energy Physics, University of Bern, Bern, Switzerland; e Laboratory of Biometry, School of Agriculture, University of Thessaly, Volos, Greece; f Division of Medical Oncology and Hematology, University and University Hospital Zurich, Zurich, Switzerland; g Diagnostic Haematology, Basel University Hospital, Basel, Switzerland; h Institut für Immunologie und Transfusionsmedizin, Universitätsmedizin Greifswald, Greifswald, Germany; i Department of Laboratory Medicine, Kantonsspital Aarau, Aarau, Switzerland; j Clinic of Medical Oncology and Hematology, Municipal Hospital Zurich Triemli, Zurich, Switzerland; k Division of Hematology and Central Hematology Laboratory, Cantonal Hospital of Lucerne and University of Bern, Switzerland; l Clinic of Hematology, Oncology Institute of Southern Switzerland, Bellinzona, Switzerland; m Department of Hematology and Central Hematology Laboratory, Inselspital, Bern University Hospital, University of Bern, Bern, Switzerland; n Division of Hematology, CHI Franciscan Medical Group, Seattle, United States; o Cantonal Hospital of St Gallen, Switzerland; p ARTORG Center for Biomedical Engineering Research, University of Bern, Bern, Switzerland; q Centre for Clinical Transfusion Medicine, University Hospital of Tübingen, Tübingen, Germany

**Keywords:** Heparin, Low-molecular-weight, Thrombocytopenia, Anticoagulants, Platelet count, Heparin-induced thrombocytopenia, Diagnosis

## Abstract

**Background:**

Diagnosing heparin-induced thrombocytopenia (HIT) at the bedside remains challenging, exposing a significant number of patients at risk of delayed diagnosis or overtreatment. We hypothesized that machine-learning algorithms could be utilized to develop a more accurate and user-friendly diagnostic tool that integrates diverse clinical and laboratory information and accounts for complex interactions.

**Methods:**

We conducted a prospective cohort study including 1393 patients with suspected HIT between 2018 and 2021 from 10 study centers. Detailed clinical information and laboratory data were collected, and various immunoassays were conducted. The washed platelet heparin-induced platelet activation assay (HIPA) served as the reference standard.

**Findings:**

HIPA diagnosed HIT in 119 patients (prevalence 8.5%). The feature selection process in the training dataset (75% of patients) yielded the following predictor variables: (1) immunoassay test result, (2) platelet nadir, (3) unfractionated heparin use, (4) CRP, (5) timing of thrombocytopenia, and (6) other causes of thrombocytopenia. The best performing models were a support vector machine in case of the chemiluminescent immunoassay (CLIA) and the ELISA, as well as a gradient boosting machine in particle-gel immunoassay (PaGIA). In the validation dataset (25% of patients), the AUROC of all models was 0.99 (95% CI: 0.97, 1.00). Compared to the currently recommended diagnostic algorithm (4Ts score, immunoassay), the numbers of false-negative patients were reduced from 12 to 6 (−50.0%; ELISA), 9 to 3 (−66.7%, PaGIA) and 14 to 5 (−64.3%; CLIA). The numbers of false-positive individuals were reduced from 87 to 61 (−29.8%; ELISA), 200 to 63 (−68.5%; PaGIA) and increased from 50 to 63 (+29.0%) for the CLIA.

**Interpretation:**

Our user-friendly machine-learning algorithm for the diagnosis of HIT (https://toradi-hit.org) was substantially more accurate than the currently recommended diagnostic algorithm. It has the potential to reduce delayed diagnosis and overtreatment in clinical practice. Future studies shall validate this model in wider settings.

**Funding:**

Swiss National Science Foundation (SNSF), and International Society on Thrombosis and Haemostasis (ISTH).

**Research in context**

**Evidence before this study**

We searched MEDLINE and EMBASE databases through the Ovid platform for journal articles presenting or validating diagnostic algorithms for heparin-induced thrombocytopenia (HIT) that utilize clinical information or heparin/platelet factor 4 immunoassay test results. Six studies proposed two-step algorithms, performing H/PF4 immunoassays serially after a positive 4Ts score. Reported sensitivities and specificities of these algorithms varied and a formal external validation is pending. Besides, some of the algorithms are complex and easy-to-use applications are currently not available.

**Added value of this study**

In this study, we developed, internally validated, and implemented an accurate and user-friendly machine learning model for the diagnosis of HIT, integrating diverse clinical and laboratory information. Our model was completely implemented on a website (https://toradi-hit.org) to facilitate its use at the bedside.

**Implications of the available evidence**

The TORADI-HIT algorithm has the potential to reduce delayed diagnosis and overtreatment in clinical practice. Future studies shall assess usability and performance in other patient populations and health care systems.

## Introduction

Heparin-induced thrombocytopenia (HIT) is a life-threatening prothrombotic disorder caused by an immune-mediated activation of platelets similar to vaccine-induced immune thrombotic thrombocytopenia.^1^, ^2^, ^3^, ^4^, ^5^, ^6^, ^7^, ^8^, ^9^, ^10^, ^11^ It affects a significant number of patients; approximately 1–3% of patients treated with heparin or up to 1 in 1500 hospitalized patients suffer from HIT.^12^, ^13^, ^14^, ^15^, ^16^, ^17^ HIT is associated with a high morbidity and mortality due to a high incidence of extensive venous and arterial thrombosis, limb loss and even death.^18^ Clinical catastrophic situations are often due to late diagnosis, undertreatment but also overtreatment.^13^ About 50% of inadequately treated patients develop severe venous and arterial thromboembolism.^15^^,^^18^, ^19^, ^20^, ^21^ Overtreatment, however, is also a major problem because therapy with alternative intravenous anticoagulants is expensive, difficult to monitor, and associated with a high rate of major bleeding complications (approximately 1% per day).^19^^,^^22^, ^23^, ^24^, ^25^, ^26^ Indeed, a significant number of patients are not diagnosed correctly in clinical practice.^19^^,^^20^^,^^22^^,^^25^^,^^27^, ^28^, ^29^, ^30^, ^31^, ^32^ Thus, a number of authors and guidelines call for new diagnostic instruments, which must not only be more accurate than conventional ones, but also easy to use.^8^^,^^28^^,^^31^^,^^33^, ^34^, ^35^

Recently, there has been meaningful progress, supporting the development of advanced diagnostic tools. HIT antibody tests were transferred to automated platforms to allow rapid determination in a 24-h operation.^36^, ^37^, ^38^ A new clinical score was proposed, and two algorithms based on a Bayesian approach were suggested.^29^^,^^33^^,^^39^^,^^40^ However, further advancements in terms of diagnostic accuracy and practicability are needed to improve diagnostic processes in clinical practice. A main drawback of today's diagnostic algorithm (Figure S1 of the supplemental material) is that limited diagnostic data (4Ts score, immunoassay result) is used *in binary form* only (positive/negative).^35^^,^^41^ In contrast, advanced machine-learning algorithms can model diverse non-linear, multivariable diagnostic information, accounting for complex hierarchical interactions.^42^^,^^43^ As a result, these models are considered to better represent the multifaceted biological interactions of the human body.^44^

Hypothesizing that machine-learning algorithms integrating diverse clinical and laboratory information could be utilized to develop a more accurate diagnostic tool than the algorithm currently recommended by the American Society of Hematology (ASH; Figure S1 of the supplemental material),^41^ we conducted a prospective cohort study and collected high-quality clinical and laboratory data. Using this information, we aimed to develop, validate, and implement an easy-to-use machine-learning prediction model for the diagnosis of HIT.

## Methods

### Study design, setting, and population

**Fig. 1:**
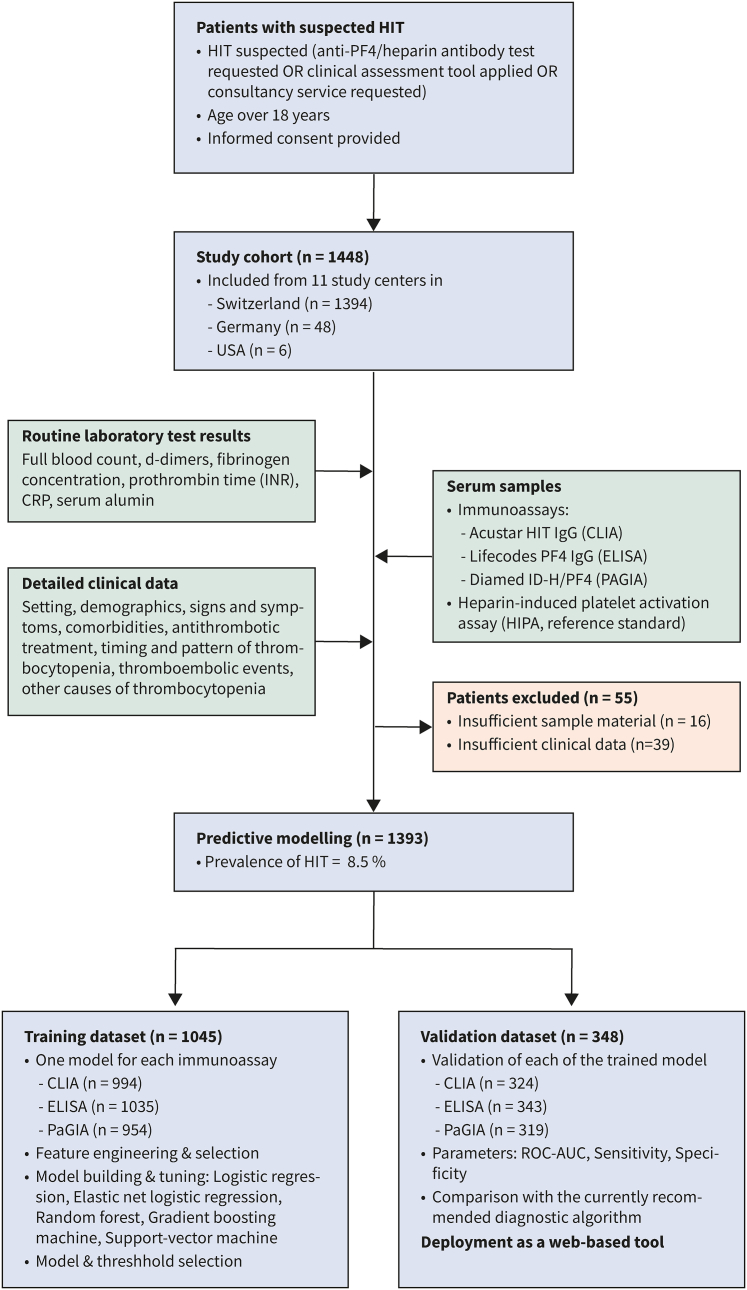
**Flow of the patients**.

The TORADI-HIT study is a prospective multicenter cohort study including consecutive patients with suspected HIT. Out of 1448 individuals included from 11 study centers in Switzerland, Germany, and the USA, 1393 were used for the current analysis (Fig. 1; Supplementary Table S1). The following inclusion criteria were applied: (1) suspected HIT ([a] anti-PF4/heparin antibody test requested, or [b] a clinical assessment tool applied, or [c] consultancy service requested), (2) age ≥18 years, and (3) informed consent provided. Exclusion criteria were (1) insufficient sample material or (2) insufficient clinical data. The study design is illustrated in Fig. 1. The well-established Swiss study group represents most University hospitals and other tertiary hospitals in Switzerland.^45^^,^^46^

Informed consent was gained either as general consent of the hospital or individual informed consent. The study protocol was approved by all ethical committees (Kantonale Ethikkommission Bern, #2017-01073) and the study was conducted according to the declaration of Helsinki. The manuscript was prepared following the TRIPOD Guidelines for the development of prediction models.^47^ The full study protocol is available upon request from the investigators.

### Study procedures and collection of data

Prespecified clinical and routine laboratory data were collected at diagnosis (suspected HIT) by specially trained study nurses using an e-CRF (REDCap database; all variables are given in the Supplementary Material). Training sessions were conducted at each study center to ensure high quality and uniform collection of data. Data were collected without knowledge of the immunoassay and HIPA test result (blinded). Follow-up data were recorded at discharge (not presented in this manuscript). Serum samples were obtained at diagnosis.

### Definition of diagnosis

The presence of HIT was defined by a positive washed-platelet functional assay, the heparin-induced platelet activation assay (HIPA; reference (gold) standard). Washed platelet assays (i.e., HIPA and serotonin release assay [SRA]) demonstrated high diagnostic sensitivity and specificity and agreement with clinical HIT.^12^^,^^34^^,^^35^^,^^41^^,^^48^, ^49^, ^50^, ^51^, ^52^, ^53^, ^54^ HIPA and SRA are both regarded as reference standards for the diagnosis of HIT by the American Society of Hematology (ASH) guidelines,^41^ the British Committee for Standards in Haematology,^53^ and many authors.^12^^,^^34^^,^^35^^,^^48^, ^49^, ^50^, ^51^^,^^55^ We decided *against the adjudication of HIT cases* by an expert panel to avoid incorporation/verification bias (clinical and laboratory variables of the prediction model are used for reference standard testing).^56^^,^^57^

### Definition of predictor variables

Based on previous publications, we selected and recorded the following potential predictor variables, whose potential values are presented in the codebook (Table S10 of the supplemental material):

#### Clinical variables

(a) degree of thrombocytopenia (10^9^/L, 24-h fall, peak, nadir),^12^^,^^58^^,^^59^ (b) timing of thrombocytopenia (according to the 4Ts score: <4 days, day 5 to day 10, >10 days),^60^, ^61^, ^62^, ^63^, ^64^ (c) presence of thrombosis (no thrombosis, suspected thrombosis, definite thrombosis, type of thrombosis),^15^^,^^60^ (d) presence of other causes of thrombocytopenia (no alternative explanation; possible other reasons; probable other reasons; detailed cause),^12^^,^^58^^,^^59^ (e) presence of major trauma,^65^ (f) presence of active cancer,^64^ (f) presence of bleeding,^66^ (g) type of anticoagulant, and (h) presence of COVID-19.

#### Laboratory variables

(a) immunoassays detecting anti-PF4/heparin antibodies (type of assay, class of antibodies, categorical result), (b) hemoglobin concentration (g/L), (c) white blood cell count (10^9^/L), (d) platelet count (10^9^/L, peak, nadir), (e) mean platelet volume (fL), (f) C-reactive protein (mg/L), (g) fibrinogen concentration (g/L), (h) serum albumin (g/L), (i) prothrombin time (s).

The following variables were additionally recorded: setting (surgery, cardiovascular surgery, internal medicine, or intensive care unit), type of hospital (primary, secondary, tertiary).

### Handling of samples and determination of laboratory tests

Residual serum samples were collected. Established in-house protocols were followed to ensure adequate preanalytical conditions and samples were frozen at −80 °C. The samples were sent on dry ice to the central laboratory, where they were treated anonymously. Three immunoassays were conducted within one week after delivery: an IgG-specific ELISA (LIFECODES IgG PF4 enhanced, Immucor, Dreieich, Germany), PaGIA (DiaMed SA, Cressier sur Morat, Switzerland), and chemiluminescent immunoassay (CLIA) AcuStar HIT-IgG (Instrumentation Laboratory, Bedford, MA, USA), determined on a BIO-FLASH® analyzer (Inova Diagnostics, San Diego, California, USA).^67^ Immunoassays were conducted according to the manufacturers' instructions, including internal and external quality controls; all assays were previously evaluated in our laboratory.^67^ Reading of immunoassay results were done blinded to HIPA results and predictor variables.

### Determination of heparin-induced platelet activation test (HIPA)

Within one week of arrival, HIPA was conducted in samples of all patients as previously described.^48^, ^49^, ^50^^,^^52^ The in-house HIPA was evaluated in a set of evaluation studies beforehand.^49^^,^^50^ The samples were analyzed with four different washed platelet donors with (a) buffer, (b) in the presence of 0.2 IU/mL low molecular weight heparin, (c) and in the presence of 100 IU/mL heparin. Platelet rich plasma (PRP) was prepared^49^^,^^50^ and the platelets were washed with the following procedure: First, the platelet pellets were resuspended at pH 6.3 using calcium- and magnesium-free Tyrode's buffer (adding glucose and apyrase). Second, at pH 7.2 the pellets were resuspended using calcium-magnesium-containing Tyrode's buffer and incubated for 45 min at 37 °C. Third, the patient samples were added to the wells of a 96-microwell plate after thawing (5 min at 37 °C) and heat-inactivation of residual thrombin (45 min at 56 °C). The platelet suspension and heparin (or buffer) was added, and the microplate was incubated for 45 min on a magnetic stirrer plate (two steel balls per well; 600 rpm). The wells were read every 5 min and it was interpreted as positive if aggregation in at least two donors occurred within 30 min in the presence of 0.2 IU/mL of heparin, but not in the presence of 100 IU/mL heparin. Each test plate included positive and negative controls.

### Statistical analysis and sample size calculation

All statistical analyses, model development and validation were done with the statistical software R, version 4.05.^68^ Descriptive statistics were calculated by HIPA test results. Predictors were presented either by median with corresponding interquartile range or by frequencies with corresponding percentages, depending on variable type. The sample size was calculated beforehand using the method by Alonzo et al. to find a difference in diagnostic test accuracy (ɑ = 0.05, two-sided, difference in specificity 0.05, 1-β = 0.9), resulting in 700 patients.^69^ The number was doubled to allow for efficient dataset splitting in algorithm building. In addition, minimum sample size requirements for predictive modelling were calculated based on recently published guidelines.^70^ Details are given in the supplementary material. Missing data were considered to be missing at random and imputed using a random forest-based algorithm.^71^

### Model development

#### Randomization of the patients

The “caret” package was used to perform data splitting and model training. The patients were randomized by HIPA status and split into a training (75%) and a validation dataset (25%) to avoid overfitting.^72^ Feature selection, model building, and hyperparameter tuning was performed in the training dataset and the internal validation in the validation dataset.

#### Feature selection

Aiming to develop a practical prediction model to be used at the bedside, trade-offs between model accuracy and usability were made. The following requirements were defined in advance: (a) a consistent quantity of features, limited to ten, considering time constraints of users (b) laboratory tests must be available in most hospitals, (c) clinical variables should be easy to collect. Feature selection was performed in phases, based on the CLIA data. First, the distribution of responses was examined. Variables with zero variance and near-zero variance (numeric values: coefficient of variation <5%; categorical values: frequency ratio >95% and unique value percentage <5%) were excluded, due to the risk of spurious results and non-informative predictors.^73^ Secondly, a backward stepwise procedure based on the Akaike information criterion was used in a logistic regression model. Additionally, a random forest model was fitted using all available predictors to extract the ten most important variables (package “randomforestexplainer”^74^). Finally, a focus group comprising all relevant stakeholders (physicians, laboratory specialists, and researchers) decided on the final selection of predictors ensuring high face validity and easy implementation.

#### Model training and hyperparameter tuning

A separate prediction model was trained for each immunoassay (CLIA, PaGIA, ELISA) because of the performance differences^37^ and because only one assay is available in most laboratories. Observations with available antibody test results were considered for model training only, resulting in different-sized data sets for each model. Five different supervised machine learning models were fitted to the data: logistic regression, elastic-net logistic regression, random forests, gradient boosting machine, and support vector machine with a polynomial kernel. Logistic regression is a statistical method for dichotomous outcome variables (disease present vs. disease not present) that models the logarithmically transformed odds of the positive event.^75^ The elastic-net logistic regression adds a penalty while solving for the coefficients to prevent overfitting.^43^^,^^76^ The random forest algorithm creates an ensemble of decision trees from bootstraps of the data set.^43^ The probability of the class is then estimated by averaging the answer of all trees. The gradient boosting machine algorithm is tree-based but focuses on the cases that are difficult to classify during training.^43^ The support vector machine algorithm draws boundaries between groups that maximize the margin between groups.^43^ A new prediction is then made from the distance to the patients in the training dataset that were closest to the border.

For preprocessing, the numerical data were normalized and a Yeo-Johnson-transformation was applied to reduce the impact of the skewness of numerical values.^43^^,^^77^ A five-times repeated, ten-fold cross-validation was used for model training.^43^ A synthetic minority oversampling technique was applied to account for the overrepresentation of HIPA-negative patients.^78^ This algorithm creates new synthetic cases based on the nearest neighbors and undersampled HIPA negative cases. At this stage, the model performance was measured with the logarithmic loss function and the model with the lowest logarithmic loss score was selected.^79^ Then, Receiver Operating Characteristic curves (ROC) were constructed for the selection of the model with the highest area under the ROC (AUC).

#### Determination of cut-off points

The cut-off point was determined in the training data set by an optimum threshold estimation using the “ThresholdROC” package. This algorithm is based on minimizing an overall cost function by attributing weights to the outcomes taking the classification rates, the disease prevalence, and the impact of the final results into account.^80^ Costs were set to penalize false negatives four times more than false positives since missed HIT cases are more serious than overtreatment.

### Model validation

Model validation was done on the validation data set. ROC and precision–recall curves were constructed, and the corresponding AUCs were calculated using the “pROC” and “PPROC” packages.^81^^,^^82^ The corresponding 95% confidence intervals (CI) were computed using the DeLong method.^83^ The sensitivity and specificity were calculated using the “epitools” package and compared to the currently recommended diagnostic algorithm (4Ts score, immunoassay; Figure S1).^41^^,^^84^ The method by Rolda'n-Nofuentes applying the Wald test along the Holm method was applied for hypothesis testing in the full dataset.^85^^,^^86^ As a sensitivity analysis, we created 250 splits for the training and validation data set and repeated model training and validation as described above.

### Implementation

To facilitate the application in clinical practice, we implemented the best-performing model for each immunoassay on an online web application accessible using a current smartphone. The “shiny” package for R was used and deployed on a Linux server running the open-source software “Shiny server” (Rstudio, Boston, MA, USA; https://www.rstudio.com/products/shiny/shiny-server/).^87^

### Role of the funding source

The funding sources had no role in the design of this study and played no role in the execution, analyses, interpretation of the data, or decision to submit results. The funders did not have access to the dataset. MN and HN had access to the dataset and made the decision to submit for publication.

## Results

### Study population and patient characteristics

Between 2018 and 2021, 1448 patients were included from 11 study centers (Fig. 1, Supplementary table S1). Sufficient clinical data and sample material was available in 1393 individuals (96.5%), which were considered for the present analysis; detailed patient characteristics are described in Table 1. HIPA was positive in 119 patients, resulting in a HIT prevalence of 8.5% (95% CI: 7.04, 9.98). The median age was 67.0 years (IQR 57.4, 75.1); 506 patients were female (36.3%). The setting was intensive care unit (ICU) in 519 patients (37.3%), cardiovascular surgery in 443 individuals (31.8%), and internal medicine in 273 patients (19.6%). COVID-19 was present in 89 patients (6.4%). Unfractionated heparin was used in 78.8% of the patients (n = 1098). Thromboembolism was present in 47.9% of HIPA-positive patients (n = 57) and 25.4% of HIPA-negative patients (n = 253). In HIPA-positive patients, the median 4T score was 5 (IQR 4, 6), and the median optical density (OD) of the anti-H/PF4 IgG ELISA was 2.5 (IQR 1.8, 3.0). In HIPA-negative patients, the median 4T score was 3 (IQR 2, 4), and the median ELISA OD was 0.1 (IQR 0.1, 0.2). Six-hundred sixty-one patients had a 4Ts score below 4 (47.45%) and 732 patients (52.54%) had a 4T score equal or greater than 4. The 4Ts score was performed by the hematology consultancy service in 91.6% of patients (n = 1276), the treating physician in 6.8% of cases (n = 95), the treating physicians and the consultancy service together in 1% of patients (n = 15) and other healthcare personal in less than 1% of patients (pharmacologist, laboratory specialist; n = 5). Discordant results between HIPA and the 4Ts score or immunoassay, respectively, are given in Table S3.

**Table 1:** Characteristics of 1′393 patients with suspected HIT included in a prospective cohort study.

	HIPA negative	HIPA positive	Overall	Missing values (n, %)
**N**	1274	119	1393	
Age - Median (IQR)	67.11 (57.7, 75.2)	64.7 (55.5, 74.5)	67.02 (57.4, 75.1)	
Male sex - n (%)	816 (64.2)	71 (59.7)	887 (63.8)	
Setting - n (%)				1 (0.1)
Post-Op general surgery or orthopedic surgery	123 (9.7)	9 (7.6)	132 (9.5)	
Post-OP cardiovascular	396 (31.1)	47 (39.5)	443 (31.8)	
Internal medicine	257 (20.2)	16 (13.4)	273 (19.6)	
ICU	479 (37.6)	40 (33.6)	519 (37.3)	
Major Trauma	4 (0.3)	6 (5.0)	10 (0.7)	
Other	14 (1.1)	1 (0.8)	15 (1.1)	
4T Score - Median (IQR)	3 (2,4)	5 (4,6)	4 (2,4)	0 (0.0)
Thrombosis present - n (%)	323 (25.4)	57 (47.9)	380 (27.3)	0 (0.0)
Deep vein thrombosis	26 (8.1)	6 (10.5)	32 (8.4)	
Pulmonary embolism	61 (18.9)	14 (24.6)	75 (19.8)	
Other venous thromboses	83 (2.58)	14 (24.6)	97 (25.6)	
Myocardial infarction	11 (3.4)	3 (5.3)	14 (3.7)	
Stroke	32 (9.9)	6 (10.5)	38 (10.0)	
Skin necrosis	8 (2.5)	1 (1.8)	9 (2.4)	
Other arterial thromboses	101 (31.4)	13 (22.8)	114 (30.1)	
Unfractionated heparin - n (%)	995 (78.1)	103 (86.6)	1098 (78.8)	0 (0.0)
Low molecular weight heparin - n (%)	553 (43.4)	50 (42.0)	603 (43.3)	0 (0.0)
Platelet nadir [10^9^/L] - Median (IQR)	60 (39, 87)	52.00 (32, 73)	59.00 (38, 86)	22 (1.6)
CRP [mg/L] - Median (IQR)	88 (34, 175)	87 (44, 146)	88 (35, 172)	86 (6.2)
CLIA [U/ml] - Median (IQR)	0 (0.0, 0.1)	10.4 (3.8, 24.6)	0.0 (0.0, 0.2)	75 (5.4)
PaGIA [titre unit] - Median (IQR)	0 (0, 1)	16 (8, 64)	0 (0, 1)	120 (8.6)
ELISA [OD] - Median (IQR)	0.1 (0.1, 0.2)	2.5 (1.8, 3.0)	0.1 (0.1, 0.3)	15 (1.1)

Abbreviations: IQR - Interquartile range, Post-op - postoperative, ICU - Intensive care unit, UFH - Unfractionated heparin, LMWH - Low-molecular-weight heparin, CRP - C-reactive protein, CLIA - chemiluminescent immunoassay, PaGIA - particle-gel immunoassay.

The heparin-induced platelet activation assay (HIPA) served as the reference standard.

### Predictors of HIT

The association between various predictor variables and HIPA status is reported in Tables 2 and S2 of the supplementary material. Statistically significant predictors were (a) degree of thrombocytopenia (odds ratio [OR]: 2.95, 95% CI: 1.98, 4.68) (b) timing of thrombocytopenia (OR: 2.81, 95% CI: 2.11, 3.80), (c) Presence of thrombosis (OR: 1.72, 95% CI: 1.40, 2.10), (d) presence of other causes of thrombocytopenia (OR: 3.88, 95% CI: 2.38, 5.37), (e) unfractionated heparin use (OR: 1.81, 95% CI: 1.08, 3.22), (f) major trauma setting (OR: 20.50, 95% CI: 5.01, 94.03), (g) WBC (OR per 10^9^/L: 1.02, 95% CI: 1.01, 1.04), (h) monocyte count (OR per 10^9^/L: 1.01, 95% CI: 1.01, 1.21), (i) platelet count at inclusion (OR per 10^9^/L: 0.99, 95% CI: 0.99, 1.00), (j) platelet nadir (OR per 10^9^/L: 0.99, 95% CI: 0.99, 1.00), (k) platelet peak (OR per 10^9^/L: 1.00, 95% CI: 1.00, 1.00), (l) IgG-CLIA (OR per U/mL: 1.56, 95% CI: 1.44, 1.70), (m) IgG-ELISA (OR per OD: 13.41, 95% CI: 9.74, 19.21), and (n) PaGIA (OR per titer unit: 1.14, 95% CI: 1.11, 1.17).

**Table 2:** Association between various potential predictor variables and HIPA status: results of the univariate logistic regression analysis.

Variables	β	OR	95% CI	p-value
Age	−0.01	0.99	0.98, 1.00	0.14
Male sex	−0.20	0.82	0.56, 1.21	0.32
Setting				
Post-op general surgery of orthopedic surgery	REF	REF	REF	REF
Post-op cardiovascular	0.48	1.62	0.81, 3.62	0.20
Internal medicine	−0.16	0.85	0.37, 2.06	0.71
ICU	0.13	1.14	0.56, 2.56	0.73
Major trauma	3.02	20.50	5.01, 94.03	<0.01
Other	−0.02	0.98	0.05, 5.79	0.98
*4T - Score*				
Degree of thrombocytopenia (per 4Ts point)	1.08	2.95	1.98, 4.68	<0.01
Timing of thrombocytopenia (per 4Ts point)	1.03	2.81	2.11, 3.80	<0.01
Presence of thrombosis (per 4Ts point)	0.54	1.72	1.40, 2.10	<0.01
Possible other causes of thrombocytopenia (per 4Ts point)	1.36	3.88	2.38, 5.37	<0.01
Type of thrombosis				
Deep vein thrombosis	REF	REF	REF	REF
Pulmonary embolism	−0.01	0.99	0.36, 3.07	0.99
Other venous thromboses	−0.31	0.73	0.26, 2.24	0.56
Myocardial infarction	0.17	1.18	0.22, 5.39	0.83
Skin necrosis	−0.61	0.54	0.03, 3.88	0.59
Stroke	−0.21	0.81	0.23, 2.88	0.74
Other arterial thromboses	−0.58	0.56	0.20, 1.71	0.28
*Clinical variables*				
Chronic thrombocytopenic disorder	−0.95	0.39	0.06, 1.26	0.19
Sepsis	−0.03	0.97	0.66, 1.41	0.87
Chemotherapy	−1.09	0.34	0.10, 0.82	0.04
Active cancer	−0.32	0.73	0.44, 1.15	0.19
COVID-19	−0.11	0.90	0.37, 1.86	0.79
Bleedings present	−0.02	0.98	0.61, 1.51	0.92
*Therapy*				
Treatment known to cause thrombocytopenia	−0.30	0.74	0.31, 1.53	0.46
Prior heparin exposure	−0.22	0.80	0.55, 1.17	0.26
Unfractionated heparin	0.59	1.81	1.08, 3.22	0.03
Low molecular weight heparin	−0.06	0.94	0.64, 1.38	0.77
Vitamin K antagonists	−0.69	0.50	0.18, 1.14	0.14
DOAC	0.19	1.21	071, 1.97	0.46
*Laboratory variables*				
Hemoglobin concentration (per g/L)	−0.01	0.99	0.98, 1.00	0.20
WBC (per 10^9^/L)	0.02	1.02	1.01, 1.04	<0.01
C-reactive protein (per mg/L)	0.00	1.00	1.00, 1.00	0.38
Monocyte count (per 10^9^/L)	1.11	1.01	1.01, 1.21	0.02
Platelet count at inclusion (per 10^9^/L)	−0.01	0.99	0.99, 1.00	<0.01
Platelet nadir (per 10^9^/L)	−0.01	0.99	0.99, 1.00	<0.01
Platelet nadir <20∗10^9^/L	−0.72	0.49	0.15, 1.19	0.17
Platelet peak (per 10^9^/L)	0.00	1.00	1.00, 1.00	0.02
Mean platelet volume (per fL)	0.00	1.00	0.88, 1.15	0.96
Prothrombin time (Quick %)	0.00	1.00	0.99, 1.00	0.25
*Immunoassays*				
CLIA (per U/mL)	0.44	1.56	1.44, 1.70	<0.01
PaGIA (titre unit)	0.13	1.14	1.11, 1.17	<0.01
ELISA (per OD)	2.60	13.41	9.74, 19.21	<0.01

Abbreviations: OR - Odds ratio, 95% CI - 95% Confidence interval.

### Predictor selection

After data splitting, 1046 patients were allocated to the training dataset and 347 to the validation dataset (detailed numbers per immunoassay are shown in Fig. 1). The results of a stepwise-backward selection, importance in a random forest, clinical significance (face validity), and ease of collection were considered for predictor selection. Logistic regression identified 13 potential predictors for model building; the results of the multivariate analysis are given in Supplementary Table S4. In the random forest algorithm based on the mean minimal depth of inclusion, the ten most important predictors were: (a) immunoassay test result (mean minimal depth [MMD]: 4.11), (b) timing of thrombocytopenia (MMD: 4.62), (c) CRP (MMD: 4.78), (d) possible other causes of thrombocytopenia (MMD: 4.81), (e) white blood cell count (MMD: 5.00), (f) monocyte count (MMD: 5.02), (g) mean platelet volume (MMD: 5.15), (h) platelet nadir (MMD: 5.15), (i) prothrombine time (MMD: 5.15), and (j) hemoglobin concentration (MMD 5.15). Details of the random forest model, diagrams and importance plots are reported in the supplementary material (Figures S2–S4). Considering ease of collection in clinical practice, avoidance of duplicate variables, and face validity, the focus group finally selected the following parameters: (a) immunoassay test result (LIFECODES IgG PF4 enhanced), (b) platelet nadir, (c) unfractionated heparin use, (d) CRP, (e) the timing of thrombocytopenia, and (f) other causes of thrombocytopenia.

### Model training and validation

**Fig. 2:**
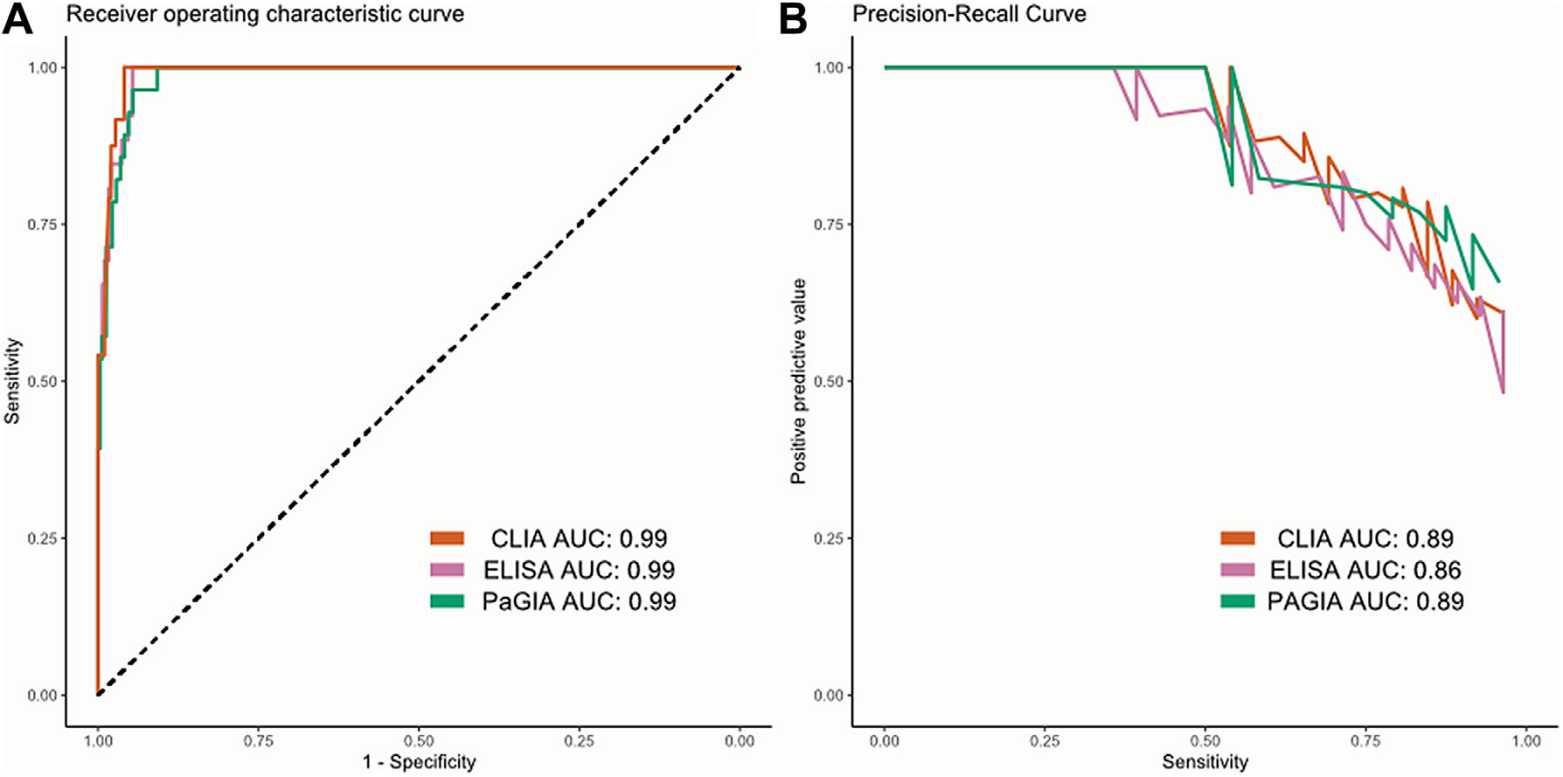
**ROC curves (A) and precision-recall curves (B) of the TORADI-HIT multivariable diagnostic prediction model in patients with suspected HIT.** Results of the validation in the validation dataset are shown. One model was developed for each immunoassay (CLIA - chemiluminescent immunoassay; PaGIA - particle gel immunoassay; ELISA - enzyme-linked immunosorbent immunoassay).

For each of the immunoassays, five different machine learning models were trained, and their hyperparameters were tuned. A table with the final hyperparameters and the corresponding logarithmic loss score is given in the Supplementary Table S4. Each of the models was then evaluated on the validation datasets; the ROC-AUC are given in Table S5 of the supplementary material. The best performing model was the support vector machine in case of the CLIA-based prediction model (AUC: 0.989, 95% CI: 0.980, 0.998), the gradient boosting machine in case of PaGIA (0.991, 95% CI: 0.982, 0.999), and the support vector machine in ELISA (0.985, 95% CI: 0.974, 0.996). The ROC and precision–recall curves are given in Fig. 2. The results of the sensitivity analysis applying 250 splits are displayed in Table S6. The median AUC for all models ranged between 0.98 and 0.99, essentially confirming the results stated above. For simpler applications, the parameters of the logistic regression models were reported in Table S7.

**Fig. 3:**
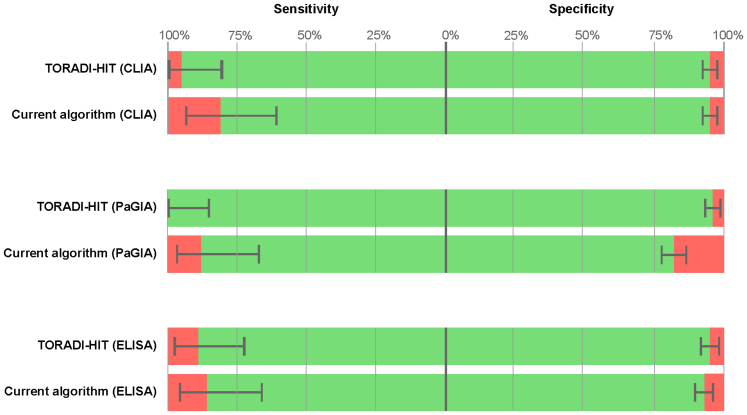
**Diagnostic accuracy of a multivariable diagnostic prediction model to be used in patients with suspected HIT.** The data were obtained in the validation dataset. Sensitivities and specificities are given in comparison to the currently recommended algorithm using one particular immunoassay (green bar, including 95% confidence intervals). The percentage of false-negative patients (left side) and false-positive patients (right side) is illustrated in red.

For each of the models, the following cut-offs were determined within the training dataset: 0.36 (CLIA), 0.41 (PaGIA), and 0.43 (ELISA). Applied to the validation dataset, the sensitivity was 96% (95% CI: 91, 97) with the CLIA, 100% (95% CI: 86, 100) with the PaGIA, and 89% (95% CI: 72, 98) with the ELISA (Table 3). The specificity was 95% (95% CI: 91, 97) with the CLIA, 95% (95% CI: 92, 97) with the PaGIA, and 95% (95% CI: 92, 97) with the ELISA. In contrast, the sensitivities, and specificities of the currently recommended algorithm (Figure S1 of the supplementary material) were 81% (95% CI: 61, 93) and 95% (95% CI: 91, 97) for CLIA, 88% (95% CI: 68, 97) and 82% (95% CI: 78, 87) for PaGIA, and 86% (95% CI: 67, 96) and 93% (95% CI: 80, 95) for ELISA. The p-value was <0.01 for all comparisons (Table S9 of the supplemental material). All diagnostic accuracy measures are reported in Table 3 and illustrated in Fig. 3. Diagnostic accuracy measures for the full dataset are given in Table S8 of the supplementary material.

**Table 3:** Diagnostic accuracy of a multivariable diagnostic prediction model for HIT as determined in the validation dataset (25% of the patients).

	n	TP	FN	TN	FP	Sensitivity (95% CI)	Specificity (95% CI)	PPV	NPV	LR+	LR−
TORADI-HIT algorithm											
CLIA (SVM)	324	25	1	282	16	96 (80, 100)	95 (91, 97)	61 (45, 76)	100 (98, 100)	17.91 (11.05, 29.02)	0.04 (0.01, 0.28)
PaGIA (GBM)	319	24	0	280	15	100 (86, 100)	95 (92, 97)	62 (45, 77)	100 (99, 100)	19.67 (12.01, 32.20)	0.00 (0.00, 0.01)
ELISA (SVM)	343	25	3	300	15	89 (72, 98)	95 (92, 97)	62 (46, 77)	99 (97, 100)	18.75 (11.26, 31.23)	0.11 (0.04, 0.33)
Current clinical algorithm											
CLIA	324	21	5	282	16	81 (61, 93)	95 (91, 97)	57 (39, 73)	98 (96, 99)	15.04 (9.01, 25.11)	0.20 (0.09, 0.45)
PaGIA	319	21	3	243	52	88 (68, 97)	82 (78, 87)	29 (19, 41)	99 (96, 100)	4.96 (3.72, 6.63)	0.15 (0.05, 0.44)
ELISA	343	24	4	292	23	86 (67, 96)	93 (89, 95)	51 (36, 66)	99 (97, 100)	11.74 (7.70, 17.89)	0.15 (0.06, 0.38)

Abbreviations: TP - true positives, FN - false negatives, TN - true negatives, FP - false positives, PPV - positive predictive value, NPV - negative predictive value, LR+ - positive likelihood ratio, LR− - negative likelihood ratio, SVM - support vector machine, GBM - gradient boosting machine.

The accuracy in the full dataset is given in Table S8 of the supplementary material. Accuracy data of the currently recommended algorithm (4Ts score + immunoassay) are given as comparison.

### Implementation

The prediction model was implemented at https://toradi-hit.org. The online calculator computes the class probability and gives the cut-offs determined in this study.

## Discussion

In a stringently executed prospective cohort study including 1393 patients with suspected HIT in 10 study centers, we developed, validated, and implemented a user-friendly machine-learning algorithm for the diagnosis of HIT. The TORADI-HIT algorithm integrates clinical characteristics, which are commonly available, and routinely used laboratory tests. Tested in the validation dataset, the performance of the model was high (ROC-AUC 0.99). Compared to the currently recommended diagnostic algorithm using 4Ts score and immunoassay test results (Figure S1), the number of false-negative patients in the whole cohort was reduced by 45.5% (ELISA), 66.6% (PaGIA), or 64.3% (CLIA). False-positive results were reduced by 72.1%, and 53.1%, respectively. However, the number of false-positive increased by 29% in the case of the CLIA. The prediction model was completely implemented on a website, which is accessible even with current smartphones (https://torad-hit.org).

Identified predictor variables are essentially in-line with previous publications. Regarding clinical characteristics, the 4Ts score was significantly associated with the presence of HIT, thus confirming previous studies.^27^^,^^62^^,^^63^^,^^88^, ^89^, ^90^, ^91^, ^92^, ^93^, ^94^, ^95^ In addition to (most of) these studies, we found that this is also the case for all individual domains (Table 2). In accordance with previous investigations, we identified unfractionated heparin use as an important predictor of HIT (Table 2).^59^^,^^96^ Interestingly, major trauma was an independent predictor of HIT. Even though this is a rather new finding, it was also observed in two previous studies.^65^^,^^97^ However, the number of individuals with major trauma was very limited in these cohorts and we decided not to consider it for model building. Several laboratory criteria were associated with the presence of HIT. As expected, the platelet nadir and the platelet count at inclusion were strongly associated with HIT (Table 2).^12^^,^^15^^,^^60^ More interestingly, we found that the platelet peak is also a predictor of HIT (Table 2). The higher the peak, the more likely HIT is, potentially excluding other causes for thrombocytopenia. We found that leucocyte count and monocyte count are associated with HIT (Table S2). This confirms previous preliminary and pre-clinical data.^98^, ^99^, ^100^ In our cohort, CRP was a strong predictor of HIT in multivariate analysis (the higher CRP, the less likely HIT; Table 2). This observation can be explained by infections and other non-HIT inflammatory conditions resulting in thrombocytopenia. As expected, immunoassay test results were strong predictors of HIT, thus confirming previous studies.^91^^,^^101^, ^102^, ^103^, ^104^, ^105^, ^106^, ^107^, ^108^, ^109^

In contrast to previous algorithms using the 4Ts score and immunoassay test results,^33^^,^^39^ we incorporated additional clinical (heparin use) and laboratory characteristics (CRP and type of immunoassay). The platelet count fall was replaced by the platelet nadir, which might be easier to determine. Overall, the prediction model comprises six items that are easy to retrieve in clinical practice. In contrast to previous algorithms, the variables were treated as numerical values (except heparin use), thus adding valuable diagnostic information. In addition, machine-learning algorithms can handle complex non-linear and hierarchical interactions present in complex biological mechanisms such as HIT.

The strengths of our investigation are that the prediction model was developed in a specially designed large clinical study, ensuring complete and accurate data. The inclusion criterion was *“patients with suspected HIT”*, accurately fitting the target population of the diagnostic prediction model. This setting facilitates a realistic assessment of diagnostic performance. Because the number of patients was relatively high, we were able to split the data into a training, and a validation data set and still obtain high statistical power. Beyond that, the prediction model was completely implemented as a functional web application that is accessible with most modern smartphones. As a significant limitation, the majority of patients were included in Switzerland, which might result in a certain degree of selection in terms of ethnicity or health care system. At the current stage of the evaluation, we were unable to conduct external validation in other settings, though this is planned. As another limitation, a set of commonly used immunoassays were employed in our study, and we cannot fully exclude that other tests perform differently. Before additional verification, the algorithm can currently only be applied to these particular assays. Of note, strict standardization of reaction conditions (e.g., incubation time and temperature, pH, buffer, substrate) ensures high consistency between laboratories (100% in case of the LIFECODES PF4 enhanced).^110^

**Fig. 4:**
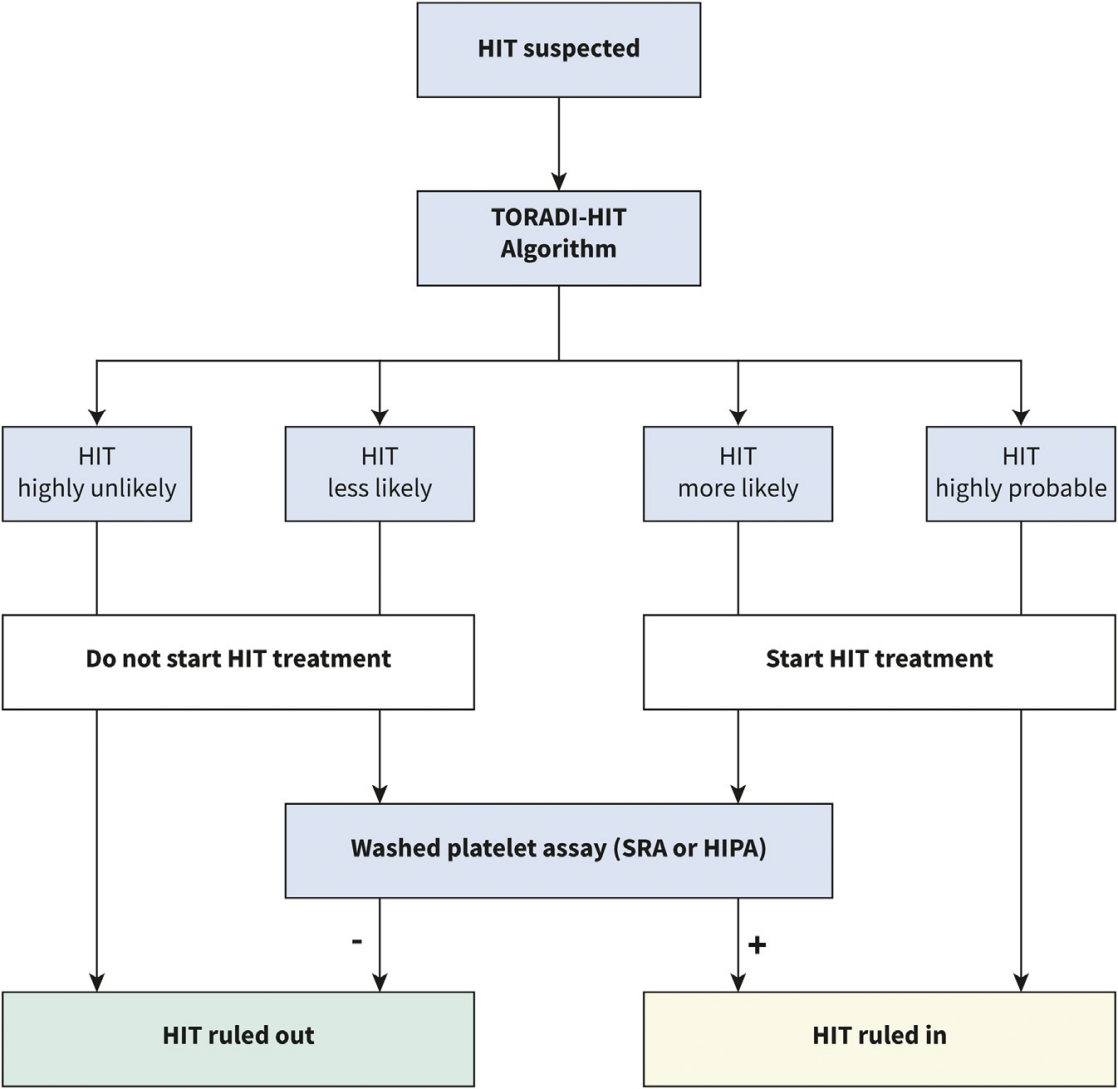
**Proposal for an adapted diagnostic workup in patients with suspected acute HIT.** The TORADI-HIT algorithm is intended to replace the current diagnostic work-up, i.e., the 4Ts score and any subsequent immunoassay. Of note, external validation studies assessing the usability and performance in other patient populations and health care systems are required before full implementation in routine clinical practice.

With the present web application, we provide a functional and easy-to-use multivariable diagnostic prediction model to be used in *patients with suspected acute HIT*. The TORADI-HIT algorithm is intended to replace the current diagnostic work-up, i.e., the 4Ts score and any subsequent immunoassay. The clinical and laboratory variables needed are available in many settings and institutions. The algorithm (www.toradi-hit.org) distinguishes very likely correct estimates (90% of HIT-positive and 90% of HIT-negative patients are in this area) from less certain assessments (10% of HIT-positive and 10% of HIT-negative individuals). The application of washed platelet assays can be considered in the latter. Fig. 4 illustrates a proposal for an adapted diagnostic workup. Considering the algorithm's ease of use and favorable clinical performance, it has the potential to reduce misdiagnosis and overtreatment in clinical practice. However, a few words of caution must be placed. First, the algorithm is not developed to rule-out other thrombocytopenic disorders or to be applied as a screening tool in unselected patients. Secondly, although the TORADI-HIT algorithm has reduced the number of clinical and thus observer-dependent variables, experienced users (such as hematology consultation teams) will provide more accurate answers than inexperienced physicians.^35^^,^^111^ Thirdly, external validation studies assessing the usability and performance in other patient populations and health care systems are required before full implementation in routine clinical practice. For this purpose, multicenter cohort studies that unselectively include consecutive patients with suspected HIT, use a locally validated washed platelet assay (SRA/HIPA) in all patients, and collect clinical data and blood samples of high, reproducible quality would be most appropriate. In addition, more rapid immunoassays such as the latex immunoassay HemosIL® HIT-Ab(PF4/H) should be added to extend the applicability of the algorithm. Furthermore, new biomarkers can potentially further improve the model's performance or reduce the complexity. Fourthly, the application of the algorithm requires the determination of one of the immunoassays used. Though the turn-around time for the chemiluminescent immunoassay in our institution is about 1 h, it is at least 24 h in case of ELISA. Therefore, until a suitable immunoassay is obtained, treating physicians are restricted to the 4Ts score. However, future algorithms that incorporate easily retrievable clinical information and ubiquitously available laboratory values could improve diagnostic workup prior to immunoassay test results.

In a specially designed prospective cohort study, we developed, validated, and implemented an easy-to-use, multivariable diagnostic prediction model. The TORADI-HIT model uses flexible machine-learning algorithms and integrates clinical characteristics, which are commonly available, and routinely used laboratory tests. In the validation dataset, the performance of the model was favorable, and the numbers of false-negative and false-positive individuals were markedly reduced compared to the currently recommended diagnostic algorithm. The prediction model was completely implemented on a website, which is accessible with current smartphones (https://toradi-hit.org/). Future studies shall assess usability and performance in other patient populations and health care systems.

## Contributors

HN wrote the analysis plan, performed the analysis, interpreted the data, and wrote the manuscript. AC and TB contributed to study design and interpretation of the data. SH, CN, and PV contributed to the analysis plan, the analysis of the data, and the interpretation. AK and SH contributed to implementation of the prediction model. JDS, DAT, AG, AM, AS, WAW, BG, JAKH, PV, LG, and TB collected data. MN designed and implemented the study, collected data, wrote the analysis plan, interpreted the data, and wrote the manuscript. All authors contributed to the interpretation of the data, revised, and approved the final manuscript.

## Data sharing statement

The dataset can be obtained upon reasonable request from the corresponding author.

## Declaration of interests

AC has served as a consultant for Synergy; has received authorship royalties for UpToDate; and his institution has received research support on his behalf from Alexion, Bayer, Novartis, Novo Nordisk, Pfizer, Sanofi, Spark, and Takeda. The institution of BG received grant support and CME support from Pfizer, Thermo Fisher Scientific, Axonlab, Sanofi, Alnylam, Bayer, BMS, Daiichi-Sankyo, Octapharma, Takeda, SOBI, Janssen, Novo Nordisk, Mitsubishi Taneba, outside of the current work. The institution of JKH received grant support, consultancy fees, or honoraria from SNSF, Baxter/Takeda, Bayer, CSL-Behring, NovoNordisk, Octapharma, Roche, SOBI, Roche, Sanofi, FOPH, and Swiss Hemophilia Society, outside of the current work. MN received research grants from Bayer Healthcare, Roche diagnostics, Siemens healthineers, Pentapharm, and Bühlmann laboratories, outside of the current work. Dr. Greinacher reports personal fees from Aspen, grants from Ergomed, grants from Boehringer Ingelheim, personal fees from Bayer Vital, grants from Rovi, grants from Sagent, personal fees from Chromatec, personal fees from Instrumentation Laboratory, grants and personal fees from Macopharma, grants from Portola, grants from Biokit, personal fees from Sanofi-Aventis, grants from Fa. Blau Farmaceutics, grants from Prosensa/Biomarin, grants and other from DRK-BSD NSTOB, grants from DRK-BSD Baden-Würtemberg/Hessen, personal fees from Roche, personal fees from GTH e.V., grants from Deutsche Forschungsgemeinschaft, grants from Deutsche Forschungsgemeinschaft, grants from Deutsche Forschungsgemeinschaft, grants from Robert-Koch-Institut, non-financial support from Veralox, grants from Dilaflor, non-financial support from Vakzine Projekt Management GmbH, grants from GIZ Else-Körner-Stiftung, grants from GIZ Else-Körner-Stiftung, non-financial support from AstraZeneca, non-financial support from Janssen Vaccines & Prevention B.V., personal fees from Takeda Pharma, personal fees from Falk Foundation e.V., grants from European Medicines Agency, outside the submitted work; In addition, AG has a patent Screening Methods for transfusion related acute lung injury (TRALI) with royalties paid to EP2321644, 18.05.2011. TM reports grant support, consultancy fees, honoraria, or support for attending meetings from DFG, Stiftung Transfusionsmedizin und Immunhämatologie e.V, DRK Blutspendedienst, Deutsche Herzstiftung, Ministerium für Wissenschaft, Forschung und Kunst Baden Würtemberg, Gesellschaft für Thrombose-und Hämostaseforschung, Berufsverband Deutscher Internisten, CoaChrom Diagnostica GmbH, Robert Bosch GmbH, Ergomed, Bayer, Bristol-Myers Squibb, Doctrina Med AG, Leo Pharma GmbH, Schöchl medical education GmbH, Mitsubishi Tanabe GmbH, Novo Nordisk GmbH, Swedish Orphan Biovitrium GmbH. All other authors declare that no conflict of interest exists.
