# Supplementary material for: A machine-learning model for reducing misdiagnosis in heparin-induced thrombocytopenia: A prospective, multicenter, observational study
Source: eClinicalMedicine. 2022 Nov 24;55:101745. doi: 10.1016/j.eclinm.2022.101745 (PMC9706528; doi:10.1016/j.eclinm.2022.101745)
Supplement: Supplementary Material [file mmc1.pdf]

Table of contents

Graphical abstract 1

Sample size calculations 2

Table S1: Inclusion of patients in 11 study centers 3

Figure S1: Currently recommended clinical algorithm 4

Table S2: Backward elimination for predictor selection: multivariate logistic regression analysis 5

Table S3: Discordant results between the 4T Score, immunoassays and the HIPA. 6

Figure S2: Distribution of minimal depth and its mean (predictor selection) 7

Figure S3: Multi-way importance plot (Mean depth of first split, number of trees in which the root is split on the variable, total number of nodes in the forest) 8

Figure S4: Multi-way importance plot (Accuracy decrease, Gini decrease, p-value) 9

Table S4: Logarithmic loss scores of the different models 10

Table S5: ROC-Curves of the different models 11

Table S6: Sensitivity analysis based on 250 trainings and validation splits 12

Table S7: Intercept and coefficients of the logistic regression models 13

Table S8: Diagnostic accuracy of a multivariable diagnostic prediction model for HIT as determined in the full cohort 14

Table S9: Statistical test between the TORADI-HIT model and the currently recommended clinical algorithm according to the method of Roldán-Nofuentes 15

Table S10: Codebook 16

Graphical abstract

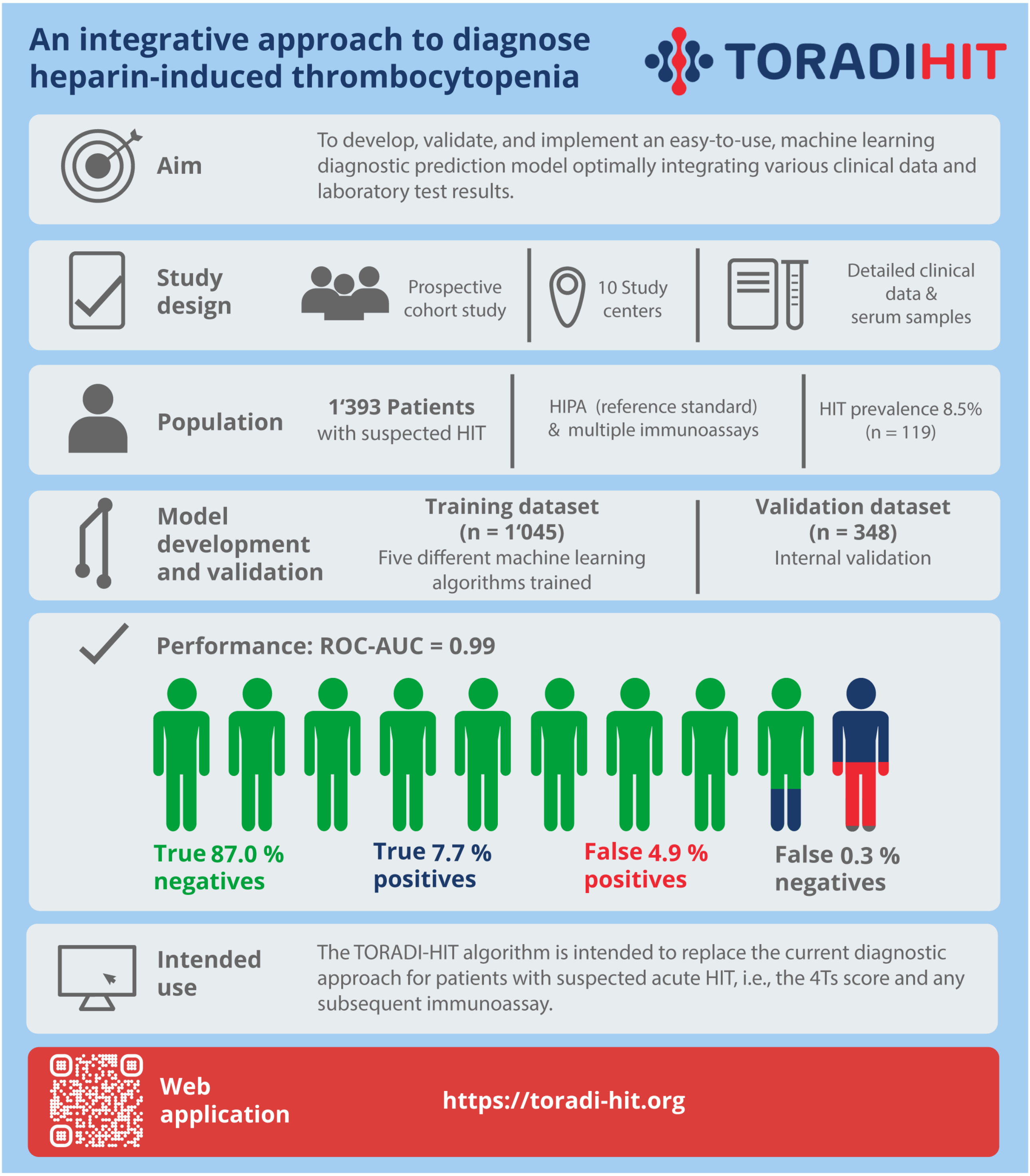

https://toradi-hit.org

Sample size calculations

1.) Minimal sample size to achieve an outcome proportion with sufficient precision (σ = 0.05)

$$n = \left(\frac{1.96}{\sigma}\right)^2 * \varphi * (1-\varphi) = \left(\frac{1.96}{0.05}\right)^2 * 0.085 * (1-0.085) = 119.51$$

2.) Target sample size and events per predictor to have a mean absolute prediction error of 0.05 was calculated using the calculator provided by van Smeden et al. The number of candidate predictors was set to 6 and the events fraction to 0.1. The minimally required sample size was 370 and the minimally required events per predictor were 6.1.

3.) Minimal sample size to achieve a shrinkage factor of 0.9

$$n = \frac{P}{(S-1) * ln(1 - \frac{R^2cs}{S})} = \frac{6}{(0.9-1) * ln(1 - \frac{0.1}{0.9})} = 509.41$$

4.) Minimal sample size to achieve a small optimism of 0.05 in the apparent R^2. With a estimated R^2 of 0.1.

$$S = \frac{0.1}{0.1 + 0.05 * 0.33} = 0.86$$

$$n = \frac{P}{(S-1) * ln(1 - \frac{R^2cs}{S})} = \frac{6}{(0.86-1) * ln(1 - \frac{0.1}{0.86})} = 346.7$$

Table S1: Inclusion of patients in 11 study centers

Institutions	Type of institution	Location	Number of patients
Inselspital, Bern University Hospital	University hospital	Bern, Switzerland	665
University Hospital Zürich	University hospital	Zürich, Switzerland	524
University Hospital Basel	University hospital	Basel, Switzerland	119
City Hospital Triemli	Tertiary teaching hospital	Zürich, Switzerland	26
University Hospital Tübingen	University hospital	Tübingen, Germany	25
Cantonal Hospital Aarau	Tertiary teaching hospital	Aarau, Switzerland	24
University Hospital Greifswald	University hospital	Greifswald, Germany	23
Cantonal Hospital Lucerne	Tertiary teaching hospital	Lucerne, Switzerland	17
Ente Ospedaliero Cantonale (EOC)	Tertiary teaching hospital	Bellinzona, Switzerland	16
Mayo Clinic Florida	Tertiary teaching hospital	Jacksonville, FL, USA	6
Cantonal Hospital St. Gallen	Tertiary teaching hospital	St. Gallen, Switzerland	3

Figure S1: Currently recommended clinical algorithm

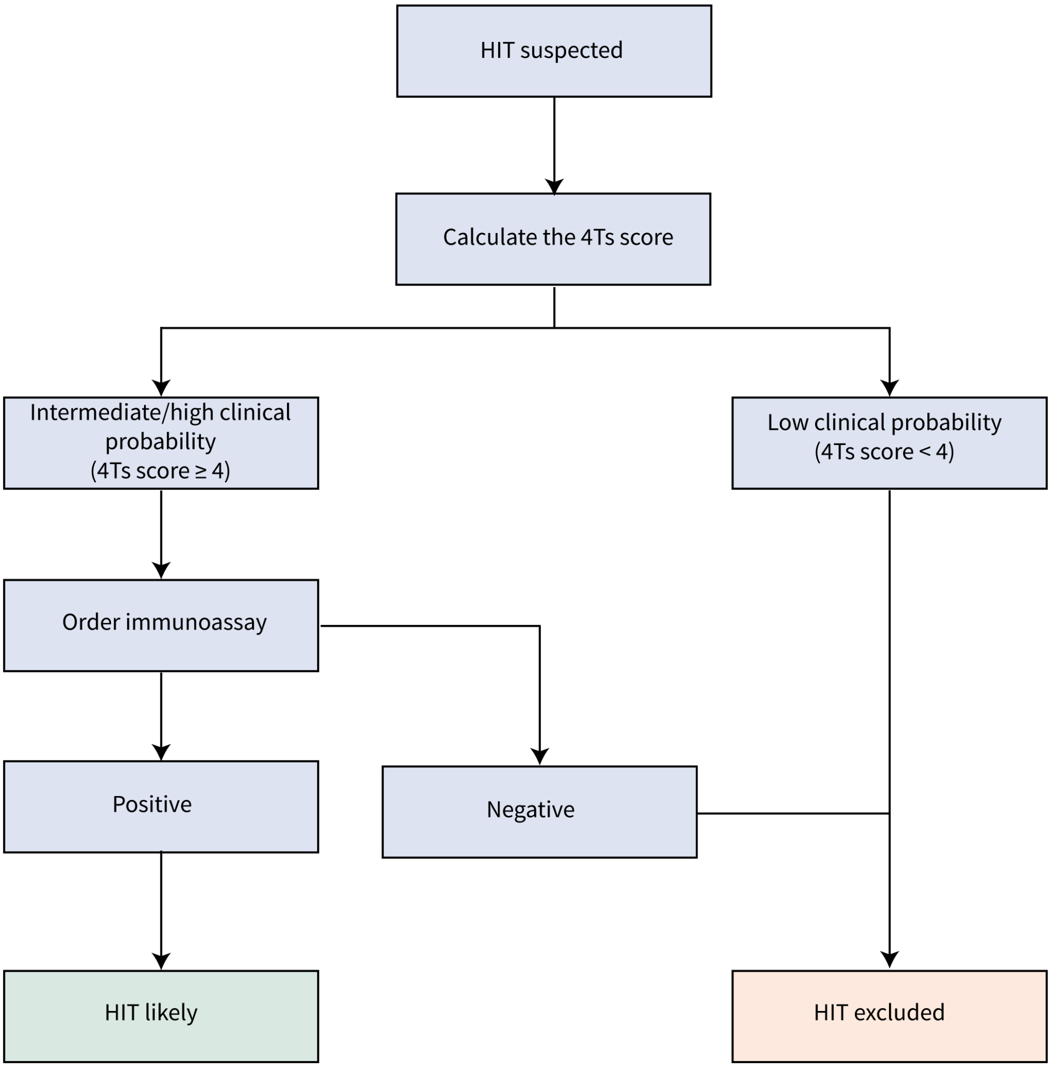

Adapted from: Cuker A, Arepally GM, Chong BH, Cines DB, Greinacher A, Gruel Y, et al. American Society of Hematology 2018 guidelines for management of venous thromboembolism: heparin-induced thrombocytopenia. Blood Advances 2018;2:3360–92. https://doi.org/10.1182/bloodadvances.2018024489.

Table S2: Backward elimination for predictor selection: multivariate logistic regression analysis

Variables	Estimate	Std. Error	p-value
Setting			
Post-Op general surgery of orthopedic surgery	0.203204	0.735915	0.78
Post-OP cardiovascular	-0.407209	0.853488	0.63
Internal medicine			
ICU	-1.064315	0.773523	0.17
Major Trauma	3.608439	1.701386	0.03
Other	-3.172307	4.760419	0.51
Degree of thrombocytopenia	0.631000	0.410318	0.12
Timing of thrombocytopenia	1.135267	0.320471	<0.001
Presence of thrombosis	1.019665	0.231622	<0.001
Possible other causes of thrombocytopenia	1.610331	0.348076	<0.001
Treatment known to cause thrombocytopenia	-1.793154	1.047000	0.09
Unfractionated heparin use	2.433287	0.991860	0.01
DOAC	0.697110	0.475644	0.14
Prior heparin exposure	-0.649232	0.397215	0.10
Leukocyte count	0.049391	0.018280	0.01
Platelet nadir	-0.016277	0.005007	<0.001
C-reactive protein	-0.006710	0.002353	<0.001
CLIA	0.365362	0.049454	<0.001

Table S3: Discordant results between the 4T Score, immunoassays and the HIPA.

Immunoassay	Test –<br>HIPA +<br>N (% of HIPA positive*)	Test +<br>HIPA –<br>N (% of HIPA negative*)
CLIA	5 (4.50 %)	67 (5.55 %)
PaGIA	1 (1.01 %)	200 (17.03 %)
ELISA	3 (2.60 %)	132 (10.45 %)
4Ts Score†	10 (8.40 %)	227 (17.82%)

*Only patients with a result for the specific immunoassay were considered for the calculation of the percentages

† A 4Ts score < 4 was considered negative and a 4Ts score ≥ 4 was considered positive

Figure S2: Distribution of minimal depth and its mean (predictor selection)

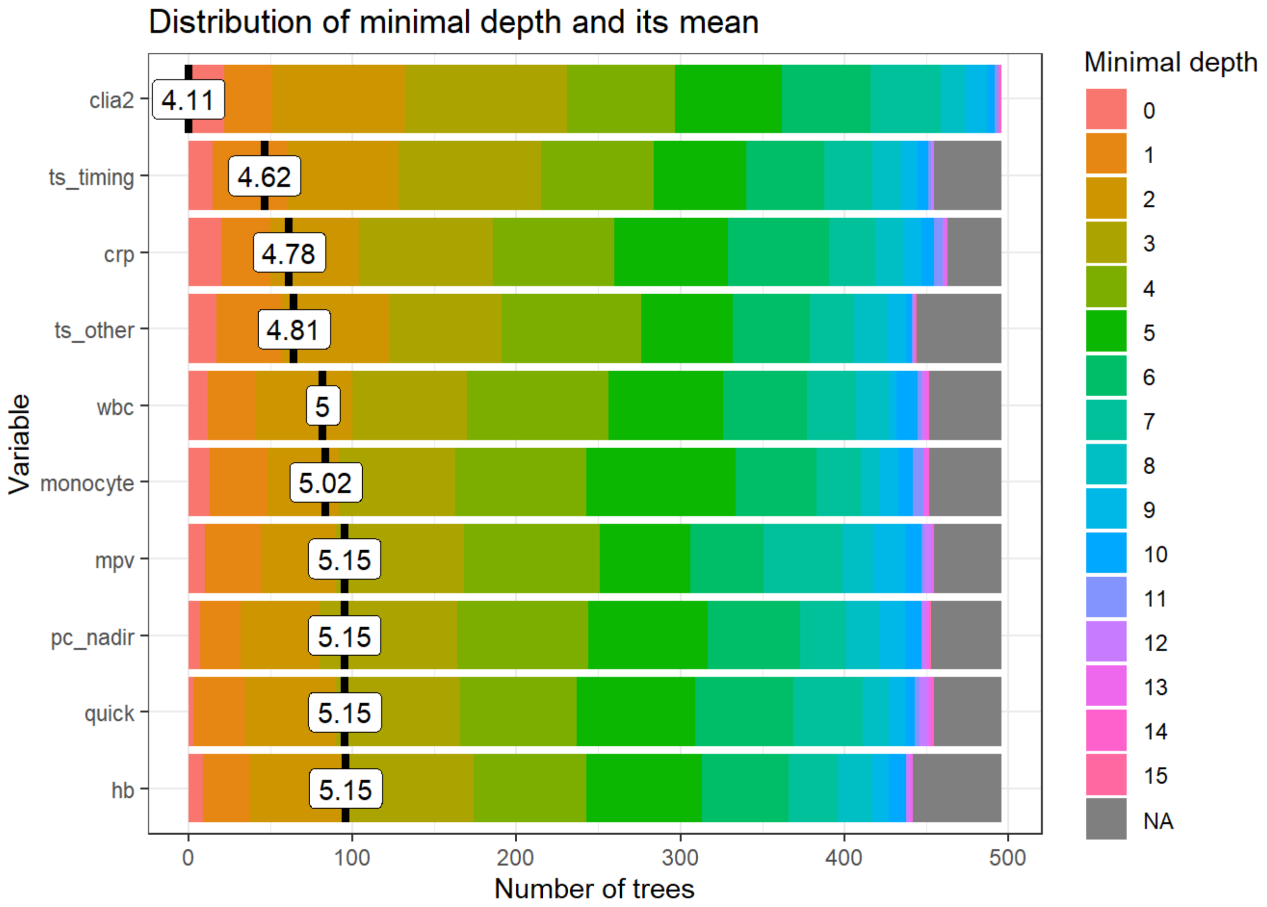

Figure S3: Multi-way importance plot (Mean depth of first split, number of trees in which the root is split on the variable, total number of nodes in the forest)

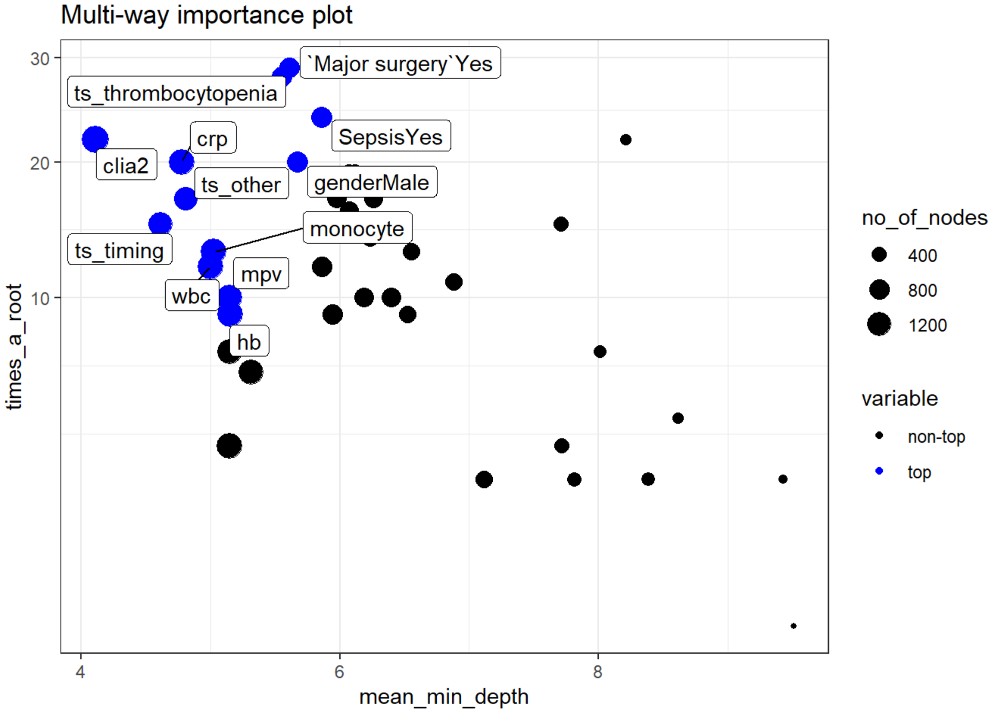

Figure S4: Multi-way importance plot (Accuracy decrease, Gini decrease, p-value)

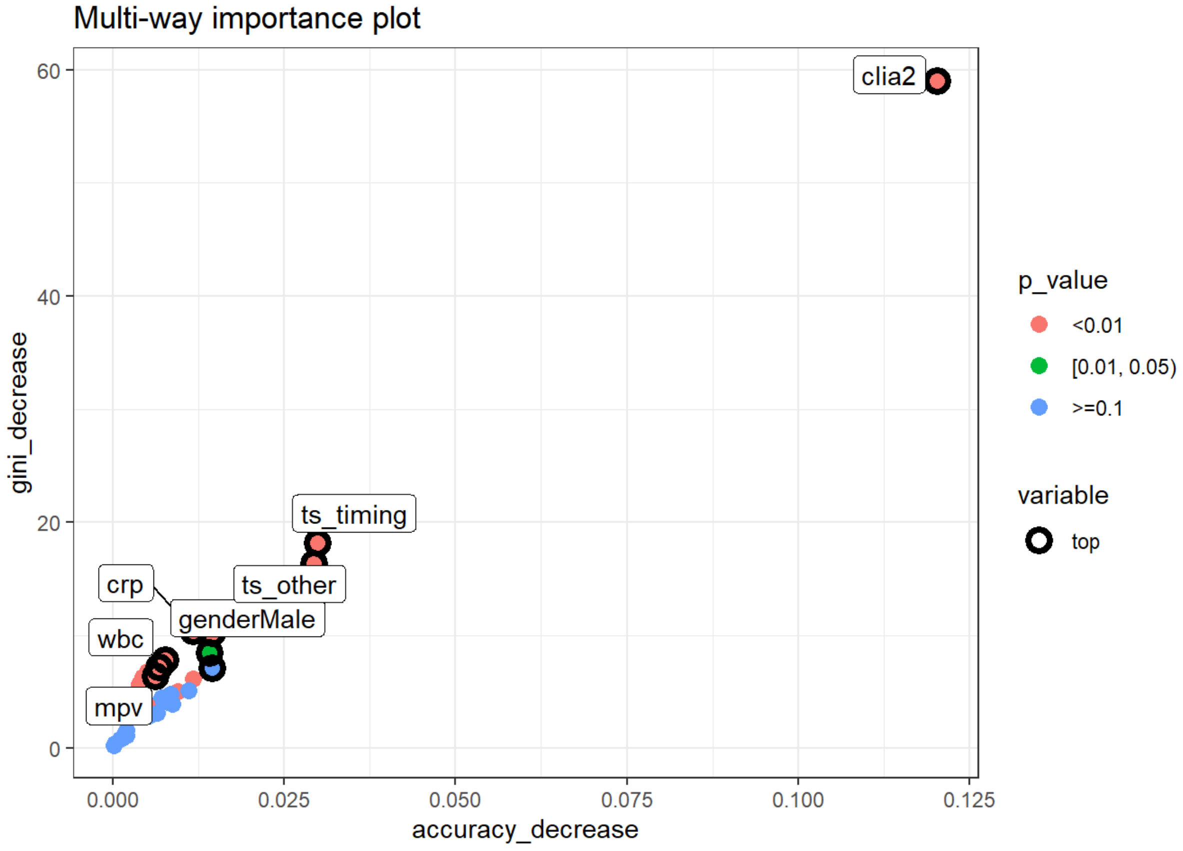

Table S4: Logarithmic loss scores of the different models

	CLIA	PaGIA	ELISA	
Logarithmic regression				-
	0.15	0.18	0.14	Logarithmic loss
Elastic net logarithmic regression	1<br>0.01	0.8<br>0.01	1<br>0.03	α<br>λ
	0.15	0.17	0.15	Logarithmic loss
Gradient boosting machine	54<br>0.075<br>3<br>5	33	100<br>0.075<br>1<br>1	No. trees,<br>shrinkage<br>interaction<br>depth,<br>Minobsinnode
	0.15	0.16	0.14	Logarithmic loss
Random forest	3	3	2	mtry
	0.20	0.19	0.15	Logarithmic loss
Support vector machine	4<br>0.001<br>2.4	10<br>0.01<br>0.7	4<br>0.01<br>0.7	Degree<br>scale<br>c
	0.15	0.16	0.13	Logarithmic loss

Table S5: ROC-Curves of the different models

	CLIA	PaGIA	ELISA
**Logarithmic regression**	0.985 (0.974, 0.996)	0.983 (0.970, 0.997)	0.981 (0.976, 0.996)
**Elastic net logarithmic regression**	0.988 (0.979, 0.998)	0.986 (0.973, 0.998)	0.984 (0.970, 0.998)
**Gradient boosting machine**	0.982 (0.970, 0.995)	0.991 (0.982, 0.999)	0.984 (0.973, 0.996)
**Random forest**	0.975 (0.960, 0.991)	0.977 (0.962, 0.992)	0.981 (0.967, 0.996)
**Support vector machine**	0.989 (0.980, 0.998)	0.988 (0.977, 0.998)	0.985 (0.974, 0.996)

Table S6: Sensitivity analysis based on 250 trainings and validation splits

	Min. AUC	Max. AUC	Median AUC	Mean AUC	SD AUC
CLIA algorithms					
Logistic regression	0.939	0.997	0.984	0.980	0.011
Elastic net logistic regression	0.936	0.996	0.984	0.980	0.012
Gradient boosting machine	0.923	0.996	0.984	0.979	0.013
Random forest	0.916	0.998	0.983	0.979	0.012
Support vector machine	0.942	0.996	0.984	0.980	0.011
PaGIA algorithms					
Logistic regression	0.923	0.996	0.980	0.978	0.012
Elastic net logistic regression	0.934	0.997	0.981	0.978	0.012
Gradient boosting machine	0.920	0.998	0.981	0.977	0.012
Random forest	0.894	0.993	0.978	0.973	0.016
Support vector machine	0.931	0.995	0.980	0.977	0.012
ELISA algorithms					
Logistic regression	0.951	0.996	0.986	0.983	0.007
Elastic net logistic regression	0.942	0.996	0.983	0.979	0.012
Gradient boosting machine	0.964	0.996	0.987	0.985	0.007
Random forest	0.953	0.995	0.985	0.983	0.007
Support vector machine	0.954	0.997	0.987	0.984	0.009

Table S7: Intercept and coefficients of the logistic regression models

	Input type	CLIA	PaGIA	ELISA
**Intercept**	-	0.3269	-0.04331	-0.3865
**Platelet nadir [10^{-9}/L]**	Numeric	-0.5630	-0.57171	-0.4413
**UFH exposure**	Categorical	0.9042	0.30374	0.8736
**CRP [mg/L]**	Numeric	-0.3665	-0.11689	-0.2231
**4 Ts – Timing of thrombocytopenia**	Numeric	0.9845	1.12405	1.1296
**4 Ts – Other causes of thrombocytopenia**	Numeric	0.5148	0.37429	0.9332
**Immunoassay test result**	Numeric	4.2541	3.83829	4.3217
**Cut-off**	-	0.39	0.42	0.42

Table S8: Diagnostic accuracy of a multivariable diagnostic prediction model for HIT as determined in the full cohort

	N	TP	FN	TN	FP	Sensitivity (95% CI)	Specificity (95% CI)	PPV	NPV	LR +	LR -
**Toradi-HIT Algorithm**											
**CLIA**	1318	106	5	1144	63	95	95	63	100	18.30	0.05
						(90, 99)	(93, 96)	(55, 70)	(99, 100)	(14.34, 23.35)	(0.02, 0.11)
**PaGIA**	1273	96	3	1111	63	97	95	60	100	18.07	0.03
						(91, 99)	(93, 96)	(52, 68)	(99, 100)	(12.01, 32.20)	(0.01, ,0.10)
**ELISA**	1378	110	6	1201	61	95	95	64	100	19.62	0.05
						(89, 98)	(94, 96)	(57, 71)	(99, 100)	(15.30, 25.15)	(0.02, 0.14)
**Current Clinical Algorithm**											
**CLIA**	1318	97	14	1157	50	87	96	66	99	21.10	0.13
						(80, 93)	(95, 97)	(58, 74)	(98, 99)	(15.94, 27.92)	(0.08, 0.21)
**PaGIA**	1273	90	9	974	200	91	83	31	99	5.34	0.11
						(83, 96)	(81, 85)	(26, 37)	(98, 100)	(4.64, 6.14)	(0.06, 0.20)
**ELISA**	1378	104	12	1175	87	90	93	54	99	13.01	0.11
						(83, 95)	(92, 94)	(47, 62)	(98, 99)	(10.52, 16.08)	(0.07, 0.19)

Table S9: Statistical test between the TORADI-HIT model and the currently recommended clinical algorithm according to the method of Roldán-Nofuentes

	Sensitvity				Specifcity				Global
	Favors	95 % CI for a sensitivity difference [%]	Wald test statistic	P-value	Favors	95 % CI for a specificty difference [%]	Wald test statistic	P-value	P-value
**CLIA**	TORADI-HIT	1.20, 12.83	6.788	< 0.01	Current algorithm	0.335, 1.816	8.961	<0.01	< 0.01
**PaGIA**	TORADI-HIT	-0.68, 12.56	3.736	< 0.01	TORADI-HIT	9.54, 13.77	118.035	< 0.01	< 0.01
**ELISA**	TORADI-HIT	-0.09. 10.26	4.682	< 0.01	TORADI-HIT	0.841, 3.273	11.368	< 0.01	< 0.01

Table S10: Codebook

Name	Data type	Labels	
pid	numeric		
gender	factor	1. Female,2. Male	
institution	factor	1. Inselspital Bern,2. University hospital Zuerich,3. University hospital Basel,4. Canton hospital Aarau,5. Canton hospital Luzerne,6. Canton hospital St. Gallen,7. EOC,8. University hospital Tuebingen,9. University hospital Greifswald,10. City hospital Triemli Zuerich 11. Mayo Clinic	
setting	factor	1. Postoperative_general_surgery_and_orthopedics,2. Postoperative_cardiac_and_vascular_surgery,3. Internal_medicine,4. ICU,5. Major_Trauma,6. Other	
observer	factor	1. Treating physician,2. Consultancy team,3. Treating physcian + Consultancy,4. Laboratory specialist,5. Pharmacist,6. Other	
tool_clinical	factor	1. 4T Score,2. Other,3. Clinical risk was not assessed	
ts_thrombocytopenia	numeric	0,1,2	
ts_timing	numeric	0,1,2	
ts_thrombosis	numeric	0,1,2	
type_thrombosis	factor	1. DVT,2. PE,3. Other VTE,4. MI,5. Stroke,6. Other ATE,7. Skin necross	
ts_other	numeric	0, 1, 2	
Chronic thrombocytopenic disorder	factor	1. No,2. Yes	
Treatment known to cause thrombocytopenia	factor	1. No,2. Yes	
Sepsis	factor	1. No,2. Yes	
Severe DIC	factor	1. No,2. Yes	
IA Device	factor	1. No,2. Yes	
Chemotherapy	factor	1. No,2. Yes	
Major surgery	factor	1. No,2. Yes	
Multiple causes	factor	1. No,2. Yes	
Other causes	factor	1. No,2. Yes	
bleeding	factor	1. No,2. Yes	
cancer	factor	1. No,2. Yes	
covid19	factor	1. No,2. Yes	
UFH	factor	1. No,2. Yes	
LMWH	factor	1. No,2. Yes	
Fondaparinux	factor	1. No,2. Yes	
Vit K Antagonist	factor	1. No,2. Yes	
DOAC	factor	1. No,2. Yes	
Dabigatran	factor	1. No,2. Yes	
dosage_clinical	factor	1. Prophylactic or intermediate dosage,2. Therapeutic dosage,3. Multiple dosages,4. Unclear	
heparin_4weeks	factor	1. No,2. Yes	
ticagrelor	factor	1. No,2. Yes	
hb	numeric		
wbc	numeric		

monocyte	numeric		
pc	numeric		
mpv	numeric		
pc_peak	numeric		
pc_nadir	numeric		
days	numeric		
d_dimers	numeric		
fibrinogen	numeric		
pt	numeric		
quick	numeric		
crp	numeric		
albumin	numeric		
elisa_igg	numeric		
pagia	numeric		
clia2	numeric		
hipa	factor	1. Negative,2. Positive	
age	numeric		
dvt	factor	1. No,2. Yes	
PE	factor	1. No,2. Yes	
skinnecrosis	factor	1. No,2. Yes	
